# Counting people inside a region-of-interest in CCTV footage with deep learning

**DOI:** 10.7717/peerj-cs.1067

**Published:** 2022-09-22

**Authors:** Bens Pardamean, Faizal Abid, Tjeng Wawan Cenggoro, Gregorius Natanael Elwirehardja, Hery Harjono Muljo

**Affiliations:** 1 Computer Science Department, BINUS Graduate Program – Master of Computer Science Program, Bina Nusantara University, Jakarta, Indonesia; 2 Bioinformatics and Data Science Research Center, Bina Nusantara University, Jakarta, Indonesia; 3 Computer Science Department, School of Computer Science, Bina Nusantara University, Jakarta, Indonesia; 4 Accounting Information Systems Program, Information Systems Department, Bina Nusantara University, Jakarta, Indonesia

**Keywords:** People counting, Deep learning, Convolutional neural networks, Region-of-Interest

## Abstract

In recent years, the performance of people-counting models has been dramatically increased that they can be implemented in practical cases. However, the current models can only count all of the people captured in the inputted closed circuit television (CCTV) footage. Oftentimes, we only want to count people in a specific Region-of-Interest (RoI) in the footage. Unfortunately, simple approaches such as covering the area outside of the RoI are not applicable without degrading the performance of the models. Therefore, we developed a novel learning strategy that enables a deep-learning-based people counting model to count people only in a certain RoI. In the proposed method, the people counting model has two heads that are attached on top of a crowd counting backbone network. These two heads respectively learn to count people inside the RoI and negate the people count outside the RoI. We named this proposed method Gap Regularizer and tested it on ResNet-50, ResNet-101, CSRNet, and SFCN. The experiment results showed that Gap Regularizer can reduce the mean absolute error (MAE), root mean square error (RMSE), and grid average mean error (GAME) of ResNet-50, which is the smallest CNN model, with the highest reduction of 45.2%, 41.25%, and 46.43%, respectively. On shallow models such as the CSRNet, the regularizer can also drastically increase the SSIM by up to 248.65% in addition to reducing the MAE, RMSE, and GAME. The Gap Regularizer can also improve the performance of SFCN which is a deep CNN model with back-end features by up to 17.22% and 10.54% compared to its standard version. Moreover, the impacts of the Gap Regularizer on these two models are also generally statistically significant (*P*-value < 0.05) on the MOT17-09, MOT20-02, and RHC datasets. However, it has a limitation in which it is unable to make significant impacts on deep models without back-end features such as the ResNet-101.

## Introduction

As closed circuit television (CCTV) has become ubiquitous in recent years, especially in developing countries (Muchtar et al., 2018), many computer vision models are rapidly adopted in practical solutions. One of the prevailing models is people counting, which is defined as an automatic system that counts the number of people captured in CCTV footage. It has been applied to numerous cases, such as crowd monitoring (Chan, Liang & Vasconcelos, 2008), scene understanding (Zhang et al., 2015; Shao et al., 2015; Zhou, Wang & Tang, 2012; Marcellino, Cenggoro & Pardamean, 2022), and smart buildings (Pardamean et al., 2021, 2019). Not only for people, automatic counting is also important in other fields such as agriculture (Alkhudaydi, Zhou & De La lglesia, 2019; Tu et al., 2020), ecological surveys (Arteta, Lempitsky & Zisserman, 2016), and traffic monitoring (Zhang et al., 2017; Ciampi et al., 2021). The implementations of automatic people counting systems were prevailing since the adoption of deep learning (LeCun, Bengio & Hinton, 2015) as people counting model, in particular convolutional neural networks (CNN) (LeCun et al., 1989). This adoption resulted in significant improvement of the state-of-the-art people counting performance.

To develop a deep-learning-based people counting model, the learning approaches that can be employed are regression approaches, which include direct and density map regression, and detection approach. Among the three mentioned approaches, direct regression is the simplest approach in which the people counting model is tasked to directly regress to the count of people in the image. The advantage of this approach is that the trained people counting model is the fastest compared to the people counting models trained with the other approaches. However, this approach restricts the model from being able to provide the coordinates of the detected people which renders it unsuitable for some video surveillance tasks or even measuring the deep learning models’ localization capability (Lian et al., 2019).

In contrast, the detection approach revolves around the use of object detection models such as Faster R-CNN (Ren et al., 2017), you only look once (YOLO) (Redmon et al., 2016), and RetinaNet (Lin et al., 2018). The object detection model is utilized to detect the object in the input image. Afterward, the detected objects are counted to produce the count of objects. This approach has better performance than the direct regression approach. However, it has a weakness in dealing with occluded heads of people, as it is often unable to detect the occluded people if the crowd is dense (Lian et al., 2019; Zhang, Shi & Chen, 2018).

**Figure 1:**
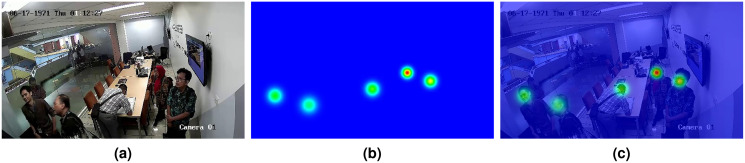
(A) A sample of input image for people counting model; (B) the corresponding density map as ground truth; (C) the input image overlaid by its density map.

Meanwhile, the density map regression approach is mainly utilized in the setting where the overlapping between people is high. This approach was first introduced by Lempitsky & Zisserman (2010) to specifically address the overlapping issue. In this strategy, a people counting model is tasked to regress a density map that is annotated with two-dimensional Gaussian distributions at each person’s head location. This density map is illustrated in Fig. 1 using jet colormap, in which pixel values are ranged from 0 to 1. The Gaussian distributions in the density map are normalized so that the sum of all values in each distribution is 1. Thus, the total people count can be obtained by summing up the values of all pixels in the density map.

**Figure 2:**
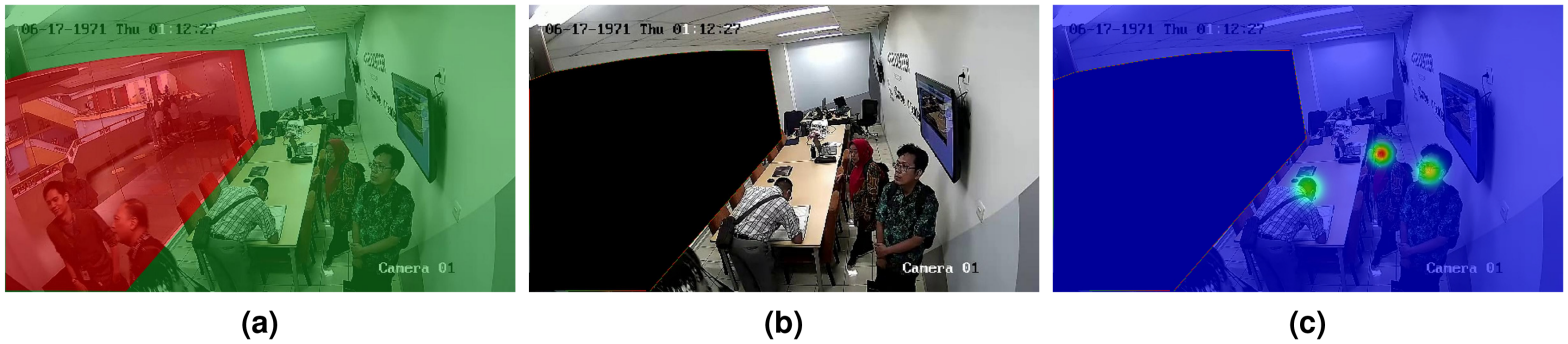
An illustration of the RoI problem in people counting, where the RoI is the area inside a room for the input image in Fig. 1. The RoI is marked in green in (A), while the area outside RoI is marked in red. The simplest solution is to cover the outside area as in (B). However, this approach also removes two persons inside the room in this case, as illustrated in (C). This is to be contrasted with the correct density map as depicted in Fig. 1C.

Oftentimes, a people counting system is required to count people only in a certain Region-of-Interest (RoI). With the density map approach, this challenge seems to be easy to be handled by covering the area outside of the RoI (Fig. 2B), so that the people in the area are not visible to the people counting model. Unfortunately, this approach is not feasible because the position of people inside and outside the RoI can be overlapped (see Fig. 2C). This problem is illustrated in Fig. 2. To overcome this issue, in this study, we propose a novel learning strategy to train a deep learning model to count people only in the predetermined RoI. The contribution of this study is twofold:

1. To design a novel learning strategy that allows a deep learning model to count people inside an RoI,
2. To develop a deep learning architecture suitable for the novel learning strategy.

## Literature review

The problem of counting people in an RoI was formally defined by Pardamean et al. (2019) in the case of developing a smart building management system. In their study, direct regression was utilized instead of the popular density map regression. In direct regression, the people counting model is tasked to straightly regress to the count of people in the CCTV footage. The performance of the models in this study was not always satisfying on several actual counts. This is expected because the direct regression approach tends to have less performance than the other approaches. To remedy the weakness of their previous study, they published a dataset that enables the development of people counting model with density map regression (Pardamean et al., 2021). The density map regression approach was chosen over the detection approach because the scene in this study may capture highly-overlapped people.

The RoI problem also emerged in the studies of crowd counting, a subset of people counting where the number of persons is massive. It was particularly seen in the WorldExpo’10 dataset (Zhang et al., 2015). Despite the issue of the overlapping position of people inside and outside of the RoI, the dataset was annotated with only the inside people. Therefore, the best people counting models on this dataset used the standard density map regression learning approach. Four models are currently the state-of-the-art models of the WorldExpo’10 in different fold of the test sets: Deep Structured Scale Integration Networks (DSSINet) (Liu et al., 2019), Modified Scale Fusion Attention Networks (M-SFANet) (Thanasutives et al., 2020), Context-Aware Networks (CAN) (Liu, Salzmann & Fua, 2019), and Perspective-Guided Convolution Networks (PGCNet) (Yan et al., 2019).

Considering the common CCTV footage view, where the perspective is linearly changed along the y-axis, the scale of people’s heads contains the information of their position. Therefore, scale-aware people counting models are potentially able to deliver better performance than the other types of people counting models. One of the most popular scale-aware people counting models is Congested Scene Recognition Networks (CSRNet) (Li, Zhang & Chen, 2018). The layers in CSRNet largely follow a typical CNN for people counting, except that five layers before the prediction layer are dilated convolution layers instead of standard convolution layers. The dilated convolution layers provide CSRNet a wider receptive field, which translates to better awareness of the perspective in its input image. The other notable scale-aware people counting model is Spatial Fully Connected Networks (SFCN) (Wang et al., 2019). It can be considered as a scale-aware model due to its unique module named DULR (Down-Up-Left-Right). This module consists of a down-up (DU) layer followed by a left-right (LR) layer. The DU and LR layers are a convolution layer with kernel size *W* × 1 and 1 × *H*, respectively. *W* and *H* are respectively the width and the height of the input feature map. The operation of the LR layer can be viewed as weighting each row in the feature map, which gives SFCN an inductive bias to encode the perspective in the input image.

Interestingly, all the state-of-the-art models of the WorldExpo’10 dataset are also scale-aware. The best model, DSSINet, is a scale-aware model because of its three subnetworks that read the same image on a different scale. On the other hand, M-SFANet used a pyramidal architecture, which has been identified as a scale-aware architecture for crowd counting (Cenggoro, Aslamiah & Yunanto, 2019). Meanwhile, CAN inserts a conductive bias for scale by downsampling the tenth layer feature map of VGG-16 (Simonyan & Zisserman, 2015) and contrasts it to the original feature map. Differently from the previously discussed models, PGCNet used a novel convolution kernel that utilizes Gaussian filtering to introduce spatial variance that can model perspective change.

## Proposed method

**Figure 3:**
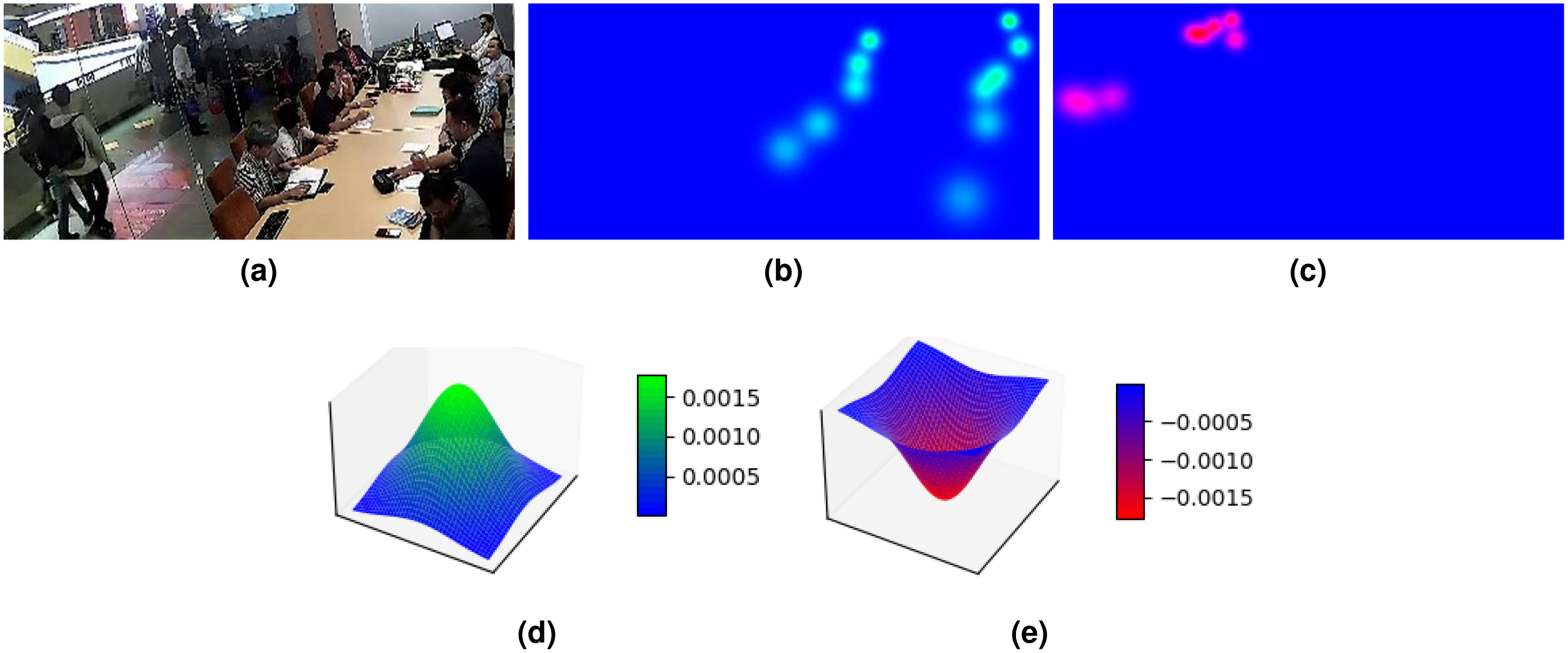
An illustration of ground truth where (A) is the input image for exclusively inside people/positive density map (B) and exclusively outside people/negative density map (C). The positive Gaussian distribution in (B) is illustrated in 3D plot in (D) and the negative Gaussian distribution in (C) is illustrated in 3D plot in (E).

For a deep-learning-based people counting model to count only people inside an RoI, we can naively supervise it to a ground truth with only people inside the RoI as illustrated in Fig. 3B. However, in this approach, the error signals from the outside people are treated the same as the background, that is, with 0 as the target in the density map. This would erase the information on the existence of the outside people. We argue that this information is beneficial for a deep learning people counting model to distinguish between people inside and outside of an RoI. Thus, a different learning strategy needs to be developed to account for this information.

One approach to incorporate the information of outside people is by annotating them as negative Gaussian distributions (Fig. 3E) in the density map, shown as red in Fig. 3C. This is to contrast with the inside people that are annotated with positive Gaussian distribution (Fig. 3D), shown as green in Fig. 3B. While this approach can let the information of outside people be learned, the developed people counting model will produce a predicted density map with an approximated negative Gaussian distribution on top of the head of outside people. Because we obtained the count by summing all pixels in the density map, the generated prediction by the model cannot be used to determine the count.

**Figure 4:**
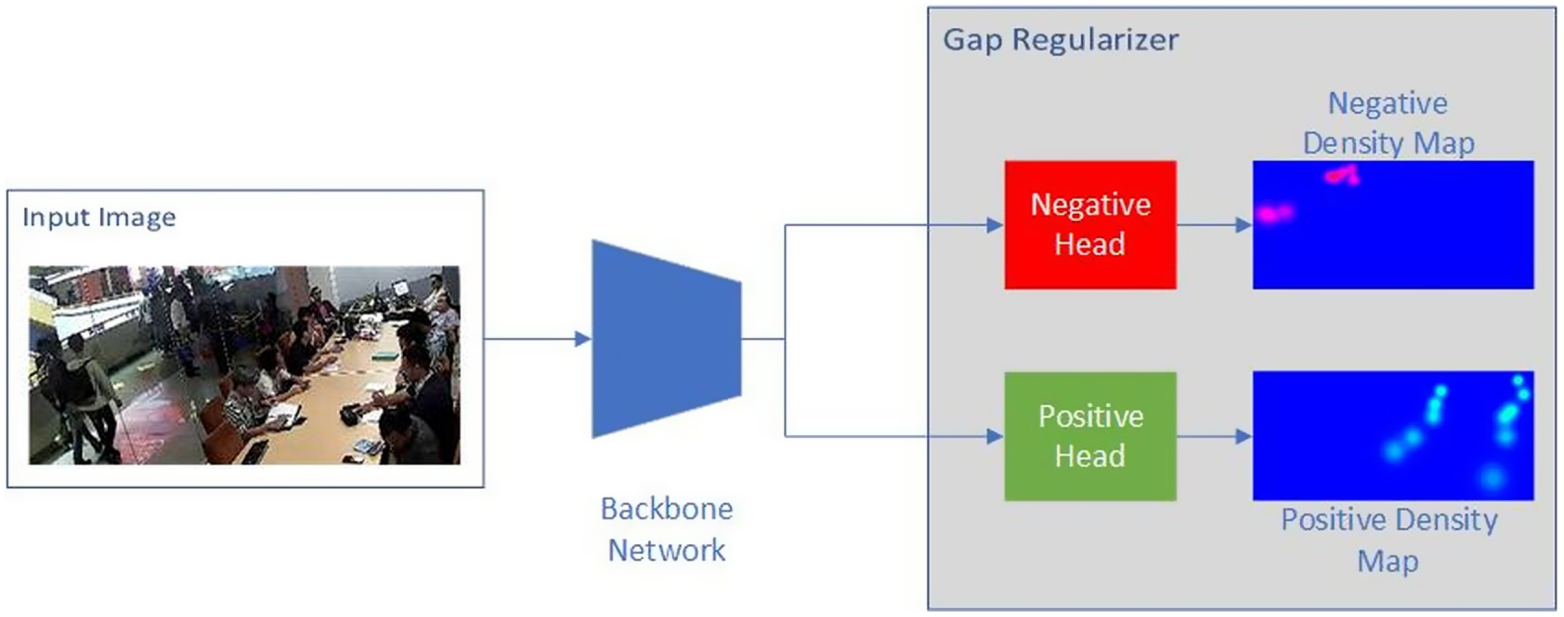
Proposed model.

To solve this problem, we proposed to separate the negative and the positive Gaussian distributions into two different density maps, as illustrated in Fig. 4. To learn from both density maps, we designed a deep learning architecture with two heads, each is supervised with the positive and negative density maps, respectively. The interaction of the positive head and negative head at the outside people location is similar to adversarial training, that is, having the two heads regress to different values. We hypothesized that this contrast training approach would establish the negative head as a regularizer that helps to improve the positive head performance. Thus, we named the twin-head architecture as Gap Regularizer, motivated by the gap between the positive and negative density map. The loss function of Gap Regularizer is defined as follows:

(1) $${{\rm {\mathcal{L}}}_{gap}} = \lambda {{\rm {\mathcal{L}}}_{pos}} + (1 - \lambda ){{\rm {\mathcal{L}}}_{neg}}$$

where ${{\rm {\mathcal{L}}}_{pos}}$ and ${{\rm {\mathcal{L}}}_{neg}}$ are respectively the loss function of the positive head and negative head. Both ${{\rm {\mathcal{L}}}_{pos}}$ and ${{\rm {\mathcal{L}}}_{neg}}$ are implemented as Mean Squared-Error (MSE), like the typical loss function of other crowd counting models. Meanwhile, $\lambda$ dictates the balance between ${{\rm {\mathcal{L}}}_{pos}}$ and ${{\rm {\mathcal{L}}}_{neg}}$. Architecturally, both the positive and negative head was a module comprising two convolutional layers with the kernel size of 1 × 1. The first layer outputs a feature map with 128 channels and the second layer outputs 1 channel that was treated as the predicted density map. The only difference is that the regular ReLU (Rectified Linear Unit) activation function was applied on the positive head whereas a negative version of it was applied on the negative head to produce the negative density maps by multiplying the ReLU results with −1. This way, the model will be trained to produce positive intermediate feature maps, and only the two heads are responsible for distinguishing the crowd inside and outside of the RoI, allowing the negative head to act as the regularizer.

The Gap Regularizer module can be attached on top of any deep-learning-based people counting models as the backbone network by first removing the original prediction layers. By having the backbone network be pretrained, the whole training process can be viewed as transfer learning, which has been proven to be beneficial in streamline computer vision tasks (Cenggoro, 2020; Girshick et al., 2014; Kornblith, Shlens & Le, 2019; Pardamean et al., 2018, 2019). Interestingly, with the design of Gap Regularizer, the information from both the people inside and outside of the RoI is able to flow to the backbone network, resulting in a backbone network that can learn to differentiate between the inside and outside people. In inference mode, the predicted density map is generated by only the positive head, so that the visible result is only to count people inside the RoI.

## Dataset

**Figure 5:**
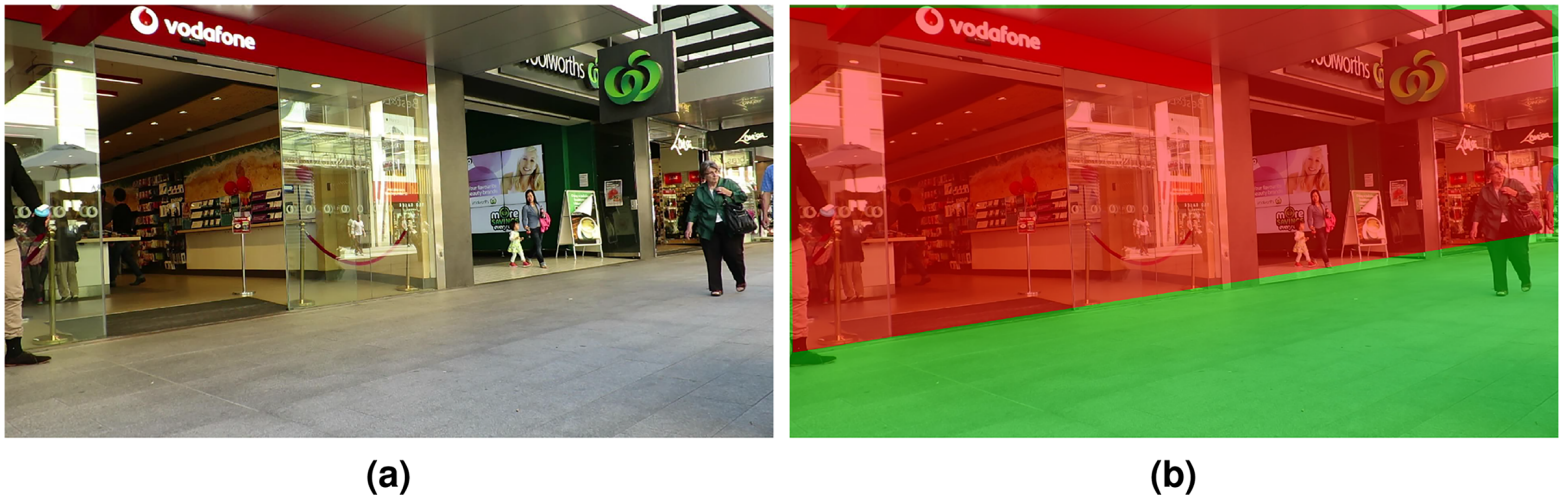
(A) A sample of an input image in the MOT17-09 dataset. (B) The defined RoI of this dataset is marked in green.

**Figure 6:**
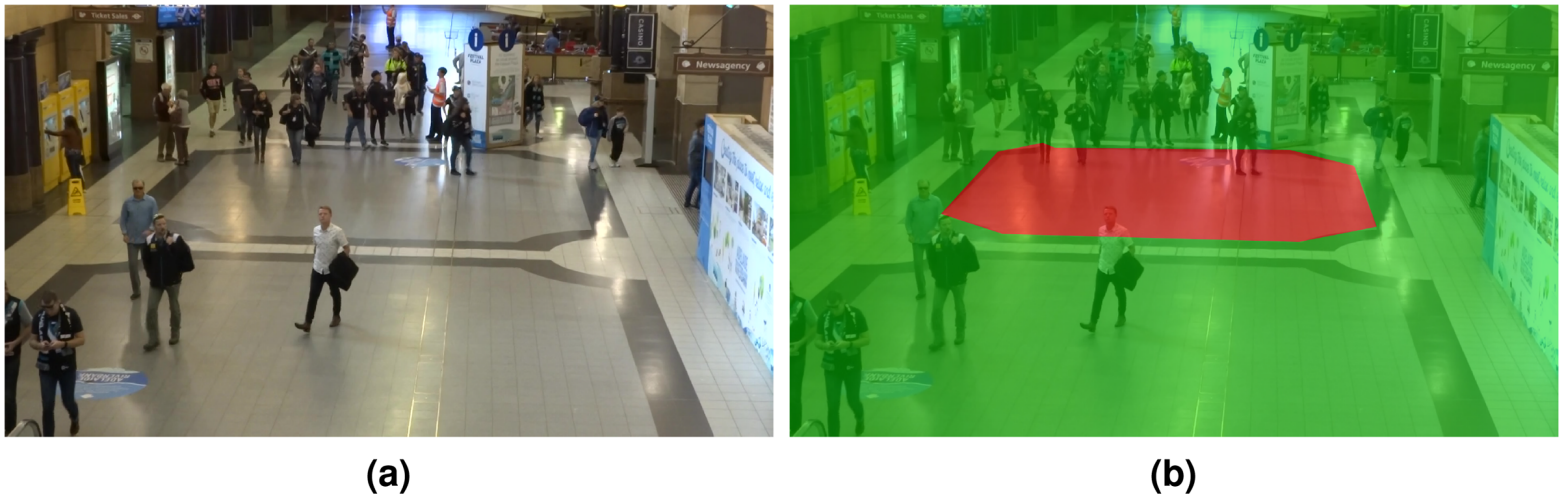
(A) A sample of an input image in the MOT20-02 dataset. (B) The defined RoI of this dataset is marked in green.

We used three datasets in this study, MOT17-09 (Milan et al., 2016; Leal-Taixé et al., 2015; Ess, Leibe & Van Gool, 2007), MOT20-02 (Dendorfer et al., 2020), and Room Human Counting (RHC) (Pardamean et al., 2021) datasets, with 525, 2,782, and 1,195 images respectively. Both MOT17-09 and MOT20-02 are subsets of the MOT17 and MOT20 datasets which are originally used for object detection of people. For this study, we split the datasets into train, validation, and test subsets, and converted all bounding boxes to 2D Gaussian distribution on top of the person’s head by assuming that all person has the proportion of average human. With this assumption, we obtained the center of the 2D Gaussian distribution by using the following formula:

(2) $${x_{gd}} = {x_{bb}} + \left(\displaystyle{w \over 2}\right)$$

(3) $${y_{gd}} = {y_{bb}} + \left(\displaystyle{h \over {16}}\right)$$

where ( ${x_{gd}}$, ${y_{gd}}$) is the coordinate of the center of the 2D Gaussian distribution, ( ${x_{bb}}$, ${y_{bb}}$) is the coordinate of the top-left corner of the bounding box, *w* is the width of the bounding box, and *h* is the height of the bounding box. The values of 2 and 16 were adopted as the humans’ heads are located at the center of the bodies’ width and the proportions are 1/8 to the bodies’ height in general (Naini et al., 2008). Afterward, we separate the annotation to people inside and outside RoI with the definition of RoI as provided in Figs. 5 and 6. A person is considered as inside the RoI if his/her feet are still within the RoI.

**Figure 7:**
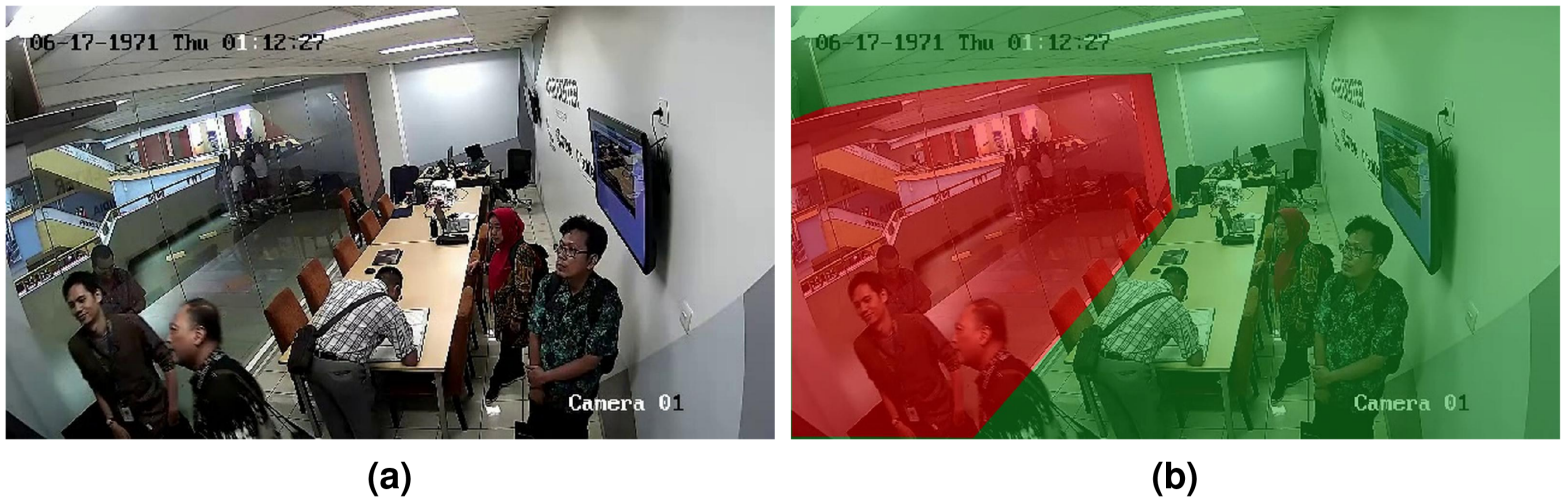
(A) A sample of an input image in the RHC dataset. (B) The defined RoI is marked in green, while the area outside RoI is marked in red.

**Figure 8:**
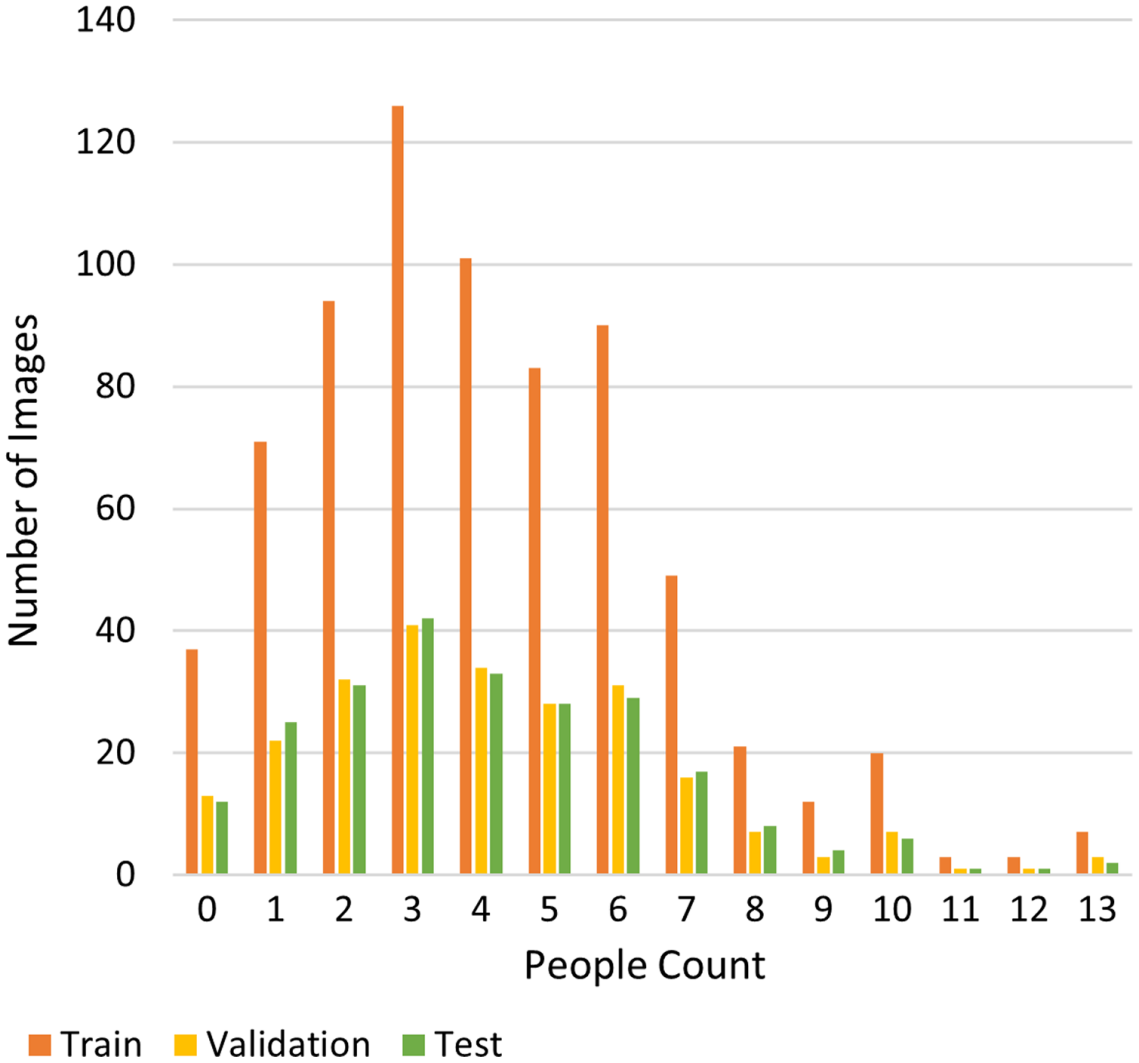
Distribution of the RHC dataset.

Meanwhile, the RHC dataset already provides the annotation of people inside an RoI and the annotation of people outside the RoI. In this case, the RoI is defined as the area of a room that was captured in the images sliced from CCTV footage. Figure 7 shows an example of an input image in the RHC dataset and the defined RoI. RHC consists of 1,195 images, split into training, validation, and test set. The distribution of this dataset is depicted in Fig. 8. The distribution of this dataset is imbalanced, which poses an additional challenge to deep learning algorithms (Cenggoro et al., 2018a; Fanny & Cenggoro, 2018; Johnson & Khoshgoftaar, 2019).

**Figure 9:**
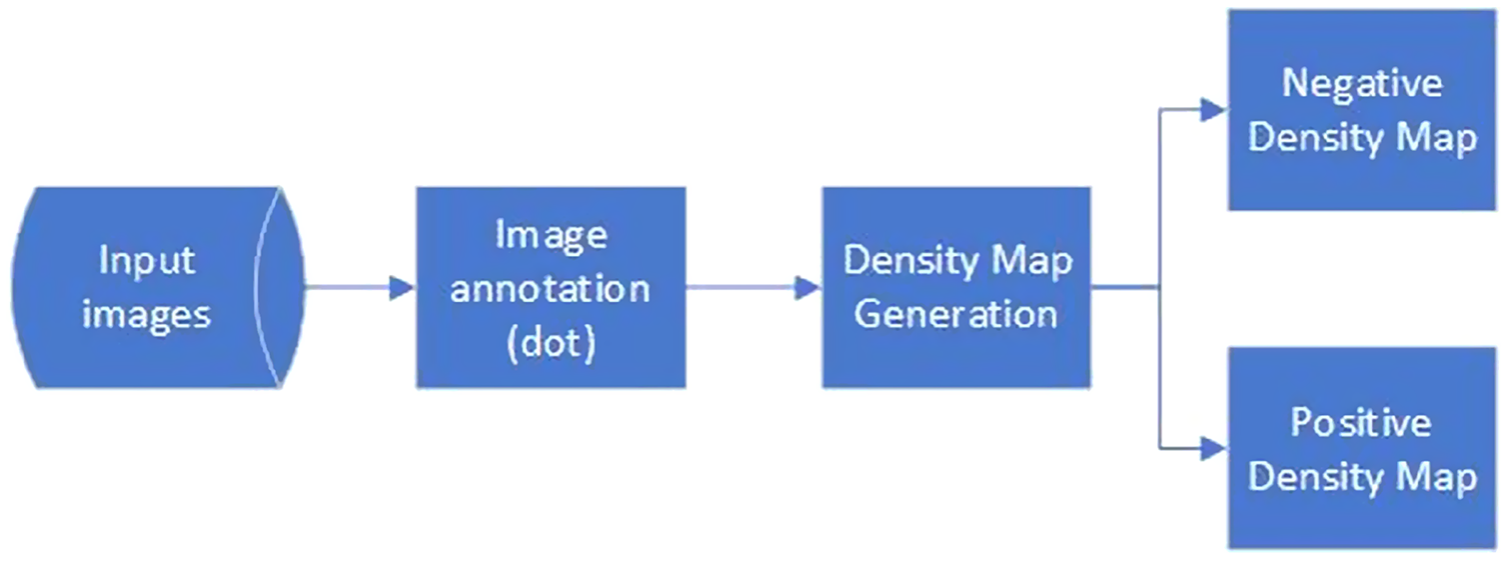
Data annotation process of the RHC dataset.

Figure 9 shows the annotation process of the RHC dataset that comprises inside and outside people density maps as the ground truth. The images were firstly annotated by putting a dot on the head of each person in the images. For each image in the dataset, this step produces dot annotations $P = \{ {P_1},{P_2}, \ldots ,{P_n}\}$, where *n* is the number of visible people in the annotated image. The dot annotations were provided by crowdworkers using a system that follows the interface recommended by Cenggoro et al. (2018b). The dot annotations were represented in a matrix with the same dimension as the annotated image, where the value of the element with the same coordinate as the center of a person’s head is 1 and the value of other elements is 0. At the same time, a separate dot annotation ${P_{in}}$ was also created for all people inside the RoI. ${P_{in}}$ was subtracted from *P* to obtain the dot annotation for all people outside the RoI.

To generate the inside and outside people density map as the ground truth of this dataset, ${P_{out}}$ and ${P_{in}}$ were multiplied with a two-dimensional Gaussian kernel with adaptive standard deviation $\sigma$ according to the location of each dot. The value of $\sigma$ represents the radius of the Gaussian kernel, meaning that its value is proportional to the size of the human heads in the images, which scales linearly with the position of the corresponding humans (Zhang et al., 2016). The calculation of the Gaussian kernel *Y* (*p*) was defined as follows:

(4) $$Y(p) = \sum\limits_{\{ p|D({p}^{\prime}) = 1\} } N (p|{p}^{\prime},{\sigma ^2}I)$$

(5) $$\sigma = max(1/3,0.063583815y - 3.09248554)$$

where *N* is a two-dimensional Gaussian Distribution function, *y* is the coordinate of the point in the y-axis.

**Figure 10:**
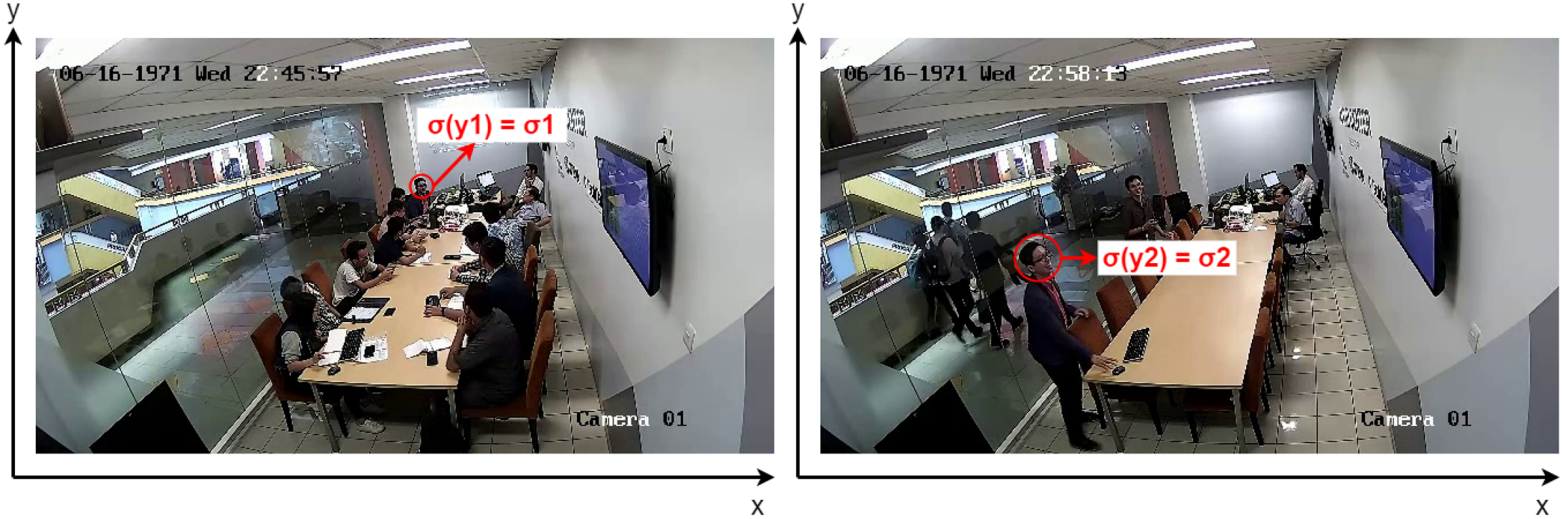
Selecting σ_1_ and σ_2_ from two images of the same person.

The constants in Eq. (5) were determined based on the ratio of the head size of the same person in a different location. Suppose that ${\sigma _1}$ and ${\sigma _2}$ are $\sigma$ of the head at the farther and nearer location from the camera respectively as illustrated by Fig. 10, the constants can be calculated as follows.

(6) $$\eqalign{& {\rm }{\sigma _1} = a{y_1} + b \cr & {\sigma _2} = a{y_2} + b}$$

(7) $$\eqalign{& a = {\sigma _1} - {\sigma _2}/{y_1} - {y_2} \cr & b = {\sigma _1} - a{y_1}}$$

where ${y_1}$ and ${y_2}$ denotes the coordinates along the y axis of the person’s heads in two images. The formulas were adopted as scaling is a linear transformation in which the value of $\sigma$ changes based on the value of *y*. Hence, the value of *a* represents the scale factor and *b* is the bias.

**Figure 11:**
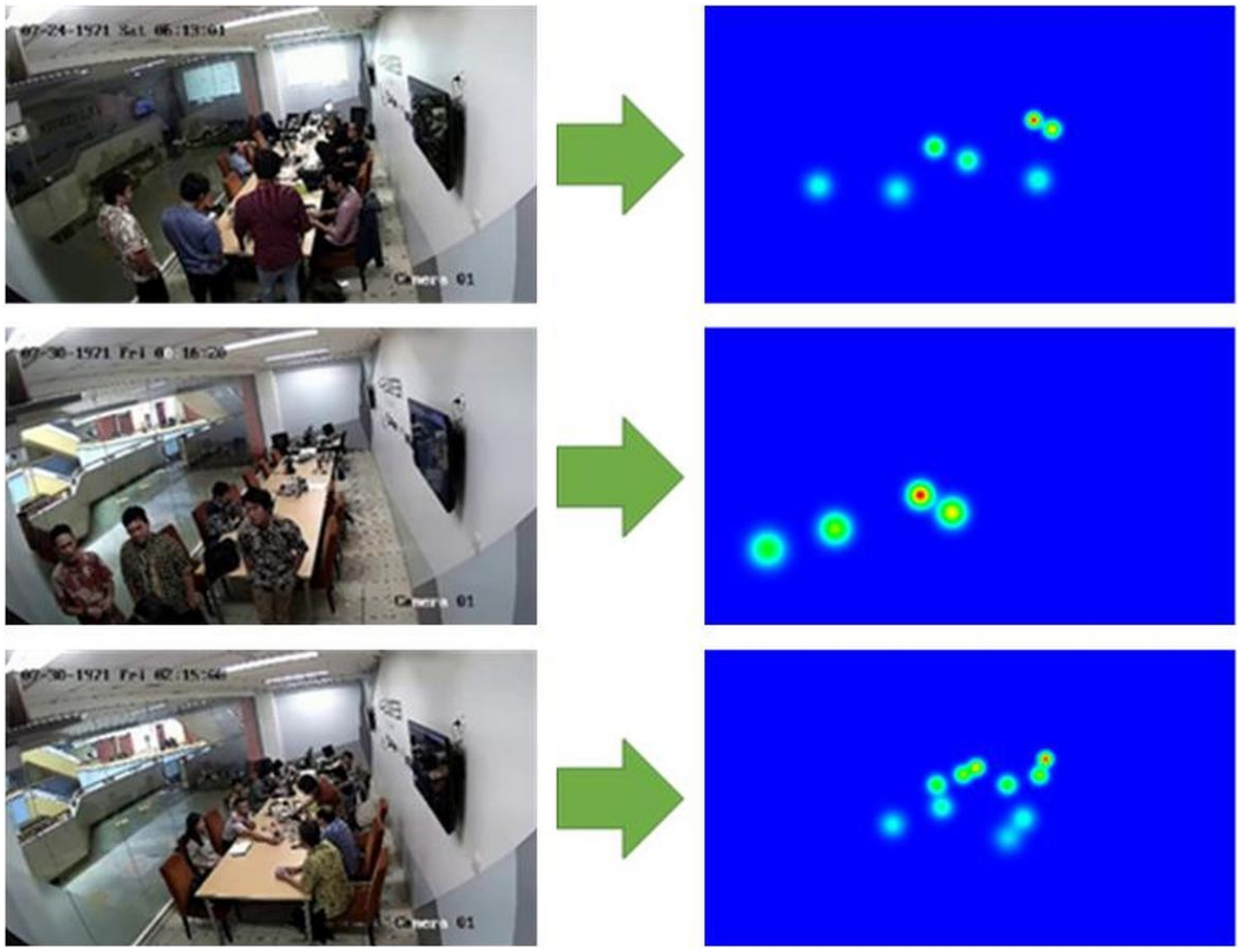
Examples of the generated density maps. The larger the y-axis coordinate of the person’s head, the wider the Gaussian distribution, because its standard deviation σ is larger. However, the peak value becomes smaller because the sum of the Gaussian distribution is normalized to 1.

Examples of the generated inside people density maps are presented in Fig. 11, where the original images are put in the left column and the density maps are put in the right column. For the MOT17-09 and MOT20-02 datasets, the value of $\sigma$ was set to 0.5 following Eq. (2) as the labels were already provided as coordinates of bounding boxes for each individual.

## Experiment setup

In this study, we considered four backbone networks. The first two networks were standard CNN networks, namely ResNet50 and ResNet101 (He et al., 2016). The other two networks were scale-aware people counting networks: CSRNet (Li, Zhang & Chen, 2018) and SFCN (Wang et al., 2019). In total, we compared eight models in this study. The first four models were standard people counting models with the four backbone networks. These models were trained with only the density map for people inside the RoI. The other four models were people counting models with the four backbone networks attached with Gap Regularizer. The two heads architecture of the Gap Regularizer modules were two 1 × 1 convolution layers. The code of all models was developed with PyTorch (Paszke et al., 2019) using C-3-Framework (Gao et al., 2019) as the codebase. All models were trained for 100 to 200 epochs using Adam optimizer (Kingma & Ba, 2015) with the learning rate of ${10^{ - 4}}$, decaying by 0.5% after every epoch. For each dataset, we tuned the $\lambda$ hyperparameter value using grid-search with the search space {0.7, 0.8, 0.9}.

To generate the inside and outside people density map as the ground truth of the RHC dataset, ${P_{out}}$ and ${P_{in}}$ were multiplied with a two-dimensional Gaussian kernel with adaptive standard deviation $\sigma$ according to the location of each dot. The calculation of the Gaussian kernel *Y*(*p*) was defined as follows:

(8) $$\ell (x,y) = L = {\{ {l_1}, \ldots ,{l_N}\} ^{\rm \top }},\quad {l_n} = {\left( {{Y_n} - Y_n^{gt}} \right)^2}$$

where *N* is the batch size, *Y* is the predicted density map, and ${Y^{gt}}$ is the ground-truth density map. *Y* and ${Y^{gt}}$ are tensors of arbitrary shapes with a total of *n* elements each. For each model, the version with the best validation loss among the 200 epochs was used for performance evaluation.

## Evaluation method

To evaluate the performance of all models, we used mean absolute error (MAE) and root mean-squared error (RMSE), which are the common metrics for crowd counting model evaluation. The MAE and RMSE are formulated with the following equation:

(9) $$RMSE = \displaystyle{1 \over {N \cdot M}}\sqrt {\sum\limits_{n = 1}^N {\sum\limits_{m = 1}^M {{{({f_{n,m}} - {y_{n,m}})}^2}} } }$$

(10) $$MAE = \displaystyle{1 \over {N \cdot M}}\sum\limits_{n = 1}^N {\sum\limits_{m = 1}^M | } {f_{n,m}} - {y_{n,m}}|$$

where (*N* * *M*) is the total pixel of the input image, with M is the number of pixels on the y-axis and N is the number of pixels on the x-axis, ${f_{n,m}}$ is the mean value of a pixel with coordinate *n*, *m* from the image generated by the model, and ${y_{n,m}}$ is the value of a pixel from the ground truth image of the input image. To test whether Gap Regularizer can improve its standard counterpart, we used Mann-Whitney U Test (Mann & Whitney, 1947), which is also known as Wilcoxon rank sum test, with MAE as the population.

We also measured the performance of all models with Grid Average Mean Error (GAME) and Structured Similarity Index Measure (SSIM) metrics to calculate the localization errors. The GAME metric is an MAE computed over patches that are defined by grids with a fixed size (Guerrero-Gómez-Olmedo et al., 2015). The formula is as follows:

(11) $$GAME\;(L)\; = \;\sum\limits_{l = 1}^{{4^L}} {\left| {{x^l}\; - \;{y^l}} \right|}$$

where *x* and *y* are the predicted and ground-truth density maps respectively. The GAME metric was calculated with 2 values of *L*, which are 1 and 2, to obtain more precise evaluation results of the models’ localization. Meanwhile, SSIM is a metric to measure the similarity between two images. In this case, SSIM is used to measure the similarity between the predicted density map *x* and the ground truth density map *y*. SSIM is calculated as follows:

(12) $$SSIM = \displaystyle{{(2{\mu _x}{\mu _y} + {c_1})(2{\sigma _{xy}} + {c_2})} \over {(\mu _x^2\mu _y^2 + {c_1})(\sigma _x^2 + \sigma _y^2 + {c_2})}}$$

where ${\mu _x}$ and ${\mu _y}$ are respectively the mean of *x* and *y*, ${\sigma _x}$ and ${\sigma _y}$ are respectively the variance of *x* and *y*, and ${\sigma _{xy}}$ is the covariance of *x* and *y*. ${c_1}$ and ${c_2}$ are constants that are calculated using the following equations.

(13) $${C_1} = {\left( {{K_1}L} \right)^2},\quad {C_2} = {\left( {{K_2}L} \right)^2}$$

with ${K_1} = 0.01$ and ${K_2} = 0.03$. *L* is the dynamic range of the pixel values. As the density maps contain floating points, the value of *L* is set to 2. In the original paper of SSIM, the values of $\mu$ and $\sigma$ are calculated through a convolution process using a 11 × 11 Gaussian window *w* with 1.5 standard deviation (Wang et al., 2004). As a result, the equations used in calculating them are set as follows.

(14) $$\matrix{ {{\rm }{\mu _x}} \hfill & { = w*x} \hfill \cr {{\sigma _x}} \hfill & { = w*{{\left( x \right)}^2} - \mu _x^2} \hfill \cr {{\sigma _{xy}}} \hfill & { = w*\left( {x \cdot y} \right) - \left( {{\mu _x} \cdot {\mu _y}} \right)} \hfill \cr }$$

As the pixels of the density maps were represented in small decimal values, the value of $\sigma$ would be very small and the SSIM will be closer to 1. This means that the luminance, contrast, and structure of the compared density maps would be almost indistinguishable. To avoid this, the density maps are first multiplied by ${10^5}$ before the SSIM is calculated. This way, small differences in the structures or contrasts can be detected. In this research, the Python Scikit-learn library was used in calculating the SSIM values.

## Results and discussion

### Training result

**Figure 12:**
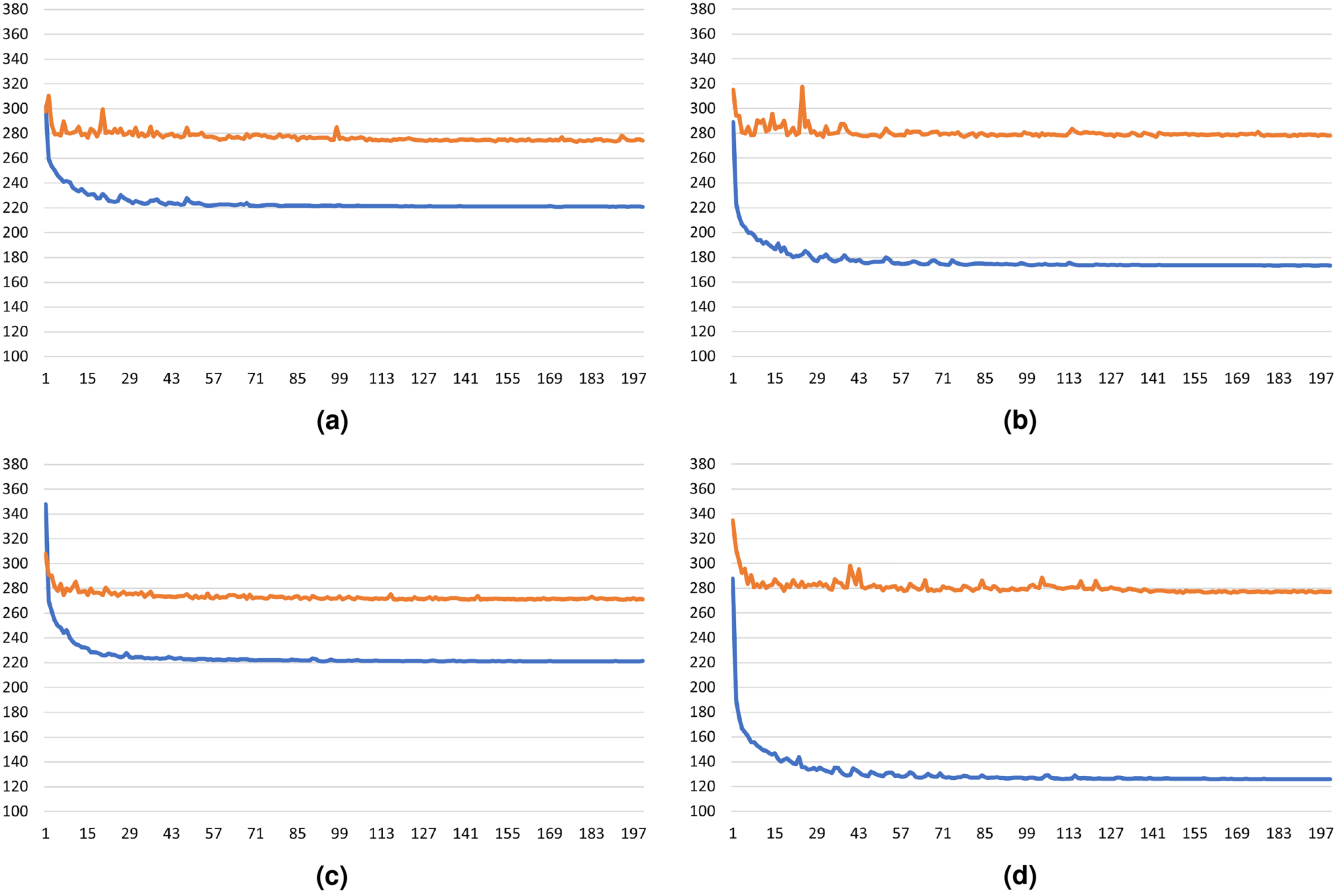
The train and validation loss plot of the: (A) ResNet-50 + Gap Regularizer model, (B) ResNet-101 + Gap Regularizer model, (C) CSRNet + Gap Regularizer model, and (D) SFCN + Gap Regularizer model.

To evaluate the generalization capability of the models with Gap Regularizer, we presented the training and validation loss plot in Figs. 12A–12D, where the y-axis is the loss value and the x-axis is the number of epochs of the training. Among the four models, CSRNet with Gap Regularizer has the best generalization capability because it is the least overfit model. This can be identified from the close gap between the training and validation loss plot. Similar results can be seen on ResNet-50, whose validation loss is slightly higher than CSRNet.

### Model performance evaluation

Table 1 summarizes the performance of all models on the test set of the MOT17-09 dataset. The experiment result shows that Gap Regularizer improves the performance of the people counting model with ResNet-50 and CSRNet as the backbone networks. ResNet-50 gains the best MAE and RMSE improvement with the use of Gap Regularizer, with 50.44% (from 2.776 to 1.376) and 45.74% (from 3.166 to 1.718) improvements respectively. The improvements of ResNet-50 are also statistically significant with the *P*-value threshold value of 0.05, unlike that of ResNet-101 and CSRNet. After using the Gap Regularizer, the MAE and RMSE of CSRNet improved by 19.68% and 21.88% on average, whereas the RMSE of ResNet-101 is slightly higher albeit the MAE is slightly lower. However, both are statitiscally insignificant. Based on the MAE and RMSE performance, the Gap Regularizer seems to degrade the people counting model with the SFCN backbone significantly. However, it is clear that the Gap Regularizer enables all models to achieve better GAME, but its impact is statistically significant only on ResNet-50. For the SSIM however, the Gap Regularizer allows all models to obtain massive improvements that are statistically significant. This indicates that the output positive density maps generated by the Gap Regularizers are far more similar to the ground-truth density maps compared to the ones produced by the standard models.

**Table 1:** The performance of all models on the MOT17-09 dataset. SM is the performance of the standard models and +GR is the performance of models with Gap Regularizer. Bold entries are the better performance between SM and +GR.

Backbone networks		ResNet-50	ResNet-101	CSRNet	SFCN
**GR** $\lambda$		0.9	0.9	0.8	0.8
	SM	2.776	1.454	0.938	**0.691**
	+GR	**1.376**	**1.446**	**0.753**	1.128
MAE	*P*-value	$6.89 \times {10^{ - 11}}$ *	$5.95 \times {10^{ - 01}}$	$5.21 \times {10^{ - 02}}$	$2.16 \times {10^{ - 07}}$ *
	SM	3.166	**1.676**	1.183	**0.868**
	+GR	**1.718**	1.781	**0.854**	1.305
RMSE	*P*-value	$6.89 \times {10^{ - 11}}$ *	$5.95 \times {10^{ - 01}}$	$5.21 \times {10^{ - 02}}$	$2.16 \times {10^{ - 07}}$ *
	SM	3.689	1.943	1.117	1.359
	+GR	**1.976**	**1.798**	**1.039**	**1.328**
GAME(1)	*P*-value	$9.91 \times {10^{ - 17}}$ *	$1.73 \times {10^{ - 01}}$	$4.13 \times {10^{ - 01}}$	$9.97 \times {10^{ - 01}}$
	SM	4.043	2.265	1.484	1.7
	+GR	**2.718**	**2.143**	**1.378**	**1.613**
GAME(2)	*P*-value	$4.64 \times {10^{ - 11}}$ *	$3.98 \times {10^{ - 01}}$	$3.64 \times {10^{ - 01}}$	$4.37 \times {10^{ - 01}}$
	SM	0.675	0.786	0.559	0.775
	+GR	**0.787**	**0.84**	**0.812**	**0.878**
SSIM	*P*-value	$5.09 \times {10^{ - 20}}$ *	$2.6 \times {10^{ - 08}}$ *	$5.86 \times {10^{ - 36}}$ *	$1 \times {10^{ - 22}}$ *
					

**Note:**

* Significant difference at *P*-value < 0.05.

Based on the results in Table 1, it can be seen that the SSIM of CSRNet experienced the greatest increase after Gap Regularizer was applied. When compared to ResNet50, the reduction in MAE, RMSE, and GAME are smaller but its improvement in SSIM is even greater. It should also be noted that the standard CSRNet achieved better MAE, RMSE, and GAME compared to ResNet50. However, the low SSIM indicate that the output density maps of the CSRNet backbone may not be too reliable, which supports the claim that metrics that are not perception-based such as MAE and RMSE may not always be reliable in perceiving visual quality (Wang et al., 2004). Therefore, it can be said that the Gap Regularizer remarkably benefits the CSRNet model, allowing it to obtain 45.11% better SSIM that is statistically significant on this dataset.

On the MOT20-02 dataset, the Gap Regularizer significantly improves the MAE and RMSE of ResNet-50, similar to the results on the MOT17-09 dataset. The MAE and RMSE improved by 33.68% and 24.25% respectively with a *P*-value of less than 0.05. For the ResNet-101 and SFCN, the results are similar to the results obtained on the MOT17-09 dataset, where the Gap Regularizer provided higher errors that are statistically insignificant. On the contrary, CSRNet with the Gap Regularizer obtained slightly better results compared to its standard model albeit the GAME(2) is insignificantly worse. Evaluation results show that the regularizer allowed ResNet-50 to obtain much better GAME and SSIM, whereas ResNet101 obtained slightly worse results. Similar to the results on the MOT17-09 dataset, the Gap Regularizer allowed CSRNet to obtain significant SSIM improvement. Such results are also visible on the SFCN backbone, where the MAE, RMSE, and GAME of the standard model are better albeit statistically insignificant, whereas the Gap Regularizer improved the model’s SSIM. It can be inferred that the Gap Regularizer generally brought positive impacts on the models on both datasets. The full results are listed in Table 2.

**Table 2:** The performance of all models on the MOT20-02 dataset. SM is the performance of the standard models and +GR is the performance of models with Gap Regularizer. Bold entries are the better performance between SM and +GR.

Backbone networks		ResNet-50	ResNet-101	CSRNet	SFCN
**GR** $\lambda$		0.7	0.9	0.9	0.9
	SM	5.756	4.015	0.707	**1.1095**
	+GR	**3.818**	**3.973**	**0.699**	1.299
MAE	*P*-Value	$4.94 \times {10^{ - 29}}$ *	$6.77 \times {10^{ - 02}}$	$9.47 \times {10^{ - 01}}$	$1.63 \times {10^{ - 01}}$
	SM	6.537	**4.691**	0.921	**1.403**
	+GR	**4.952**	4.987	**0.911**	1.816
RMSE	*P*-Value	$4.94 \times {10^{ - 29}}$ *	$6.77 \times {10^{ - 02}}$	$9.47 \times {10^{ - 01}}$	$1.63 \times {10^{ - 01}}$
	SM	6.436	**4.136**	1.264	**1.73**
	+GR	**5.067**	4.477	**1.198**	1.872
GAME(1)	*P*-Value	$1.96 \times {10^{ - 20}}$ *	$1.43 \times {10^{ - 01}}$	$3.56 \times {10^{ - 02}}$ *	$2.95 \times {10^{ - 01}}$
	SM	8.068	**4.525**	**1.724**	**2.265**
	+GR	**6.477**	5.408	1.788	2.44
GAME(2)	*P*-Value	$1.93 \times {10^{ - 30}}$ *	$7.12 \times {10^{ - 10}}$ *	$3.35 \times {10^{ - 01}}$	$1.39 \times {10^{ - 01}}$
	SM	0.587	**0.815**	0.53	0.806
	+GR	**0.679**	0.796	**0.787**	**0.82**
SSIM	*P*-Value	$8.92 \times {10^{ - 176}}$ *	$1.41 \times {10^{ - 22}}$ *	$3.19 \times {10^{ - 183}}$ *	$3.19 \times {10^{ - 183}}$ *
					

**Note:**

* Significant difference at *P*-value < 0.05.

On the RHC dataset, different phenomena are observed as the Gap Regularizer improved the MAE, RMSE, and GAME for SFCN as detailed in Table 3. However, the differences in MAE and RMSE are statistically insignificant for the ResNet-50 and CSRNet models. On the SFCN, the MAE and RMSE got reduced by 17.22% and 10.54% respectively and the reduction is statistically significant on the Mann-Whitney test with the *P*-value threshold of 0.05. The improvement of GAME are also observed in the 3 model backbones, with CSRNet obtaining statistically significant improvement by using the Gap Regularizer which indicates that the impact on CSRNet and SFCN are generally the largest for the RHC dataset. Meanwhile, even though the performance of ResNet-101 on the RHC dataset is generally lower than the standard model, the difference is not statistically significant. Unlike the results on both the MOT17-09 and MOT20-02 dataset listed in, the SSIM is statistically insignificant for the models with ResNet and SFCN backbones using Gap Regularizer on the RHC dataset. However, the SSIM of CSRNet improved from 0.276 to 0.963, a phenomenon that can also be observed on the other datasets. These results imply that the Gap Regularizer is capable of significantly improving the performance of CSRNet in all datasets.

**Table 3:** The performance of all models on the RHC dataset. SM is the performance of the standard models and +GR is the performance of models with Gap Regularizer. Bold entries are the better performance between SM and +GR.

Backbone networks		ResNet-50	ResNet-101	CSRNet	SFCN
**GR** $\lambda$		0.7	0.8	0.7	0.9
	SM	**0.224**	**0.198**	0.237	0.235
	+GR	0.228	0.228	**0.234**	**0.194**
MAE	*P*-Value	$1.45 \times {10^{ - 1}}$	$1.62 \times {10^{ - 01}}$	$1.49 \times {10^{ - 01}}$	$2.16 \times {10^{ - 02}}$ *
	SM	0.445	**0.398**	**0.349**	0.45
	+GR	**0.426**	0.436	0.366	**0.403**
RMSE	*P*-Value	$1.45 \times {10^{ - 1}}$	$1.62 \times {10^{ - 01}}$	$1.49 \times {10^{ - 01}}$	$2.16 \times {10^{ - 02}}$ *
	SM	0.347	**0.325**	0.367	0.364
	+GR	**0.333**	0.333	**0.339**	**0.316**
GAME(1)	*P*-Value	$7.23 \times {10^{ - 01}}$	$8.17 \times {10^{ - 01}}$	$1.93 \times {10^{ - 02}}$ *	$1.8 \times {10^{ - 01}}$
	SM	0.374	**0.343**	0.421	0.381
	+GR	**0.355**	0.35	**0.36**	**0.339**
GAME(2)	*P*-Value	$7.21 \times {10^{ - 01}}$	$8.53 \times {10^{ - 01}}$	$4.18 \times {10^{ - 04}}$ *	$3.34 \times {10^{ - 01}}$
	SM	**0.964**	0.9647	0.276	**0.966**
	+GR	0.963	**0.96475**	**0.963**	0.963
SSIM	*P*-Value	$6.98 \times {10^{ - 01}}$	$8.05 \times {10^{ - 01}}$	$8.73 \times {10^{ - 80}}$ *	$3.49 \times {10^{ - 01}}$

**Note:**

* Significant difference at *P*-value < 0.05.

With the fact that ResNet-50 and CSRNet are respectively the smallest and the second-smallest backbone network (see Table 4), we can conclude that Gap Regularizer is more effective on models with smaller backbones. This might be caused by the fact that Gap Regularizer is attached only on top of the backbone network. Therefore, Gap Regularizer has more effect on more shallow models because of the nature of backpropagation that has the tendency to assign smaller gradients on earlier layers as the model goes deeper. On both the MOT17-09 and MOT20-02, it significantly reduced the MAE, RMSE, and GAME of ResNet-50. For CSRNet, the Gap Regularizer dramatically improved the SSIM on all datasets and the difference compared to the standard models are statistically significant, which further supports the idea that the Gap Regularizer can yield more impact on more shallow models as CSRNet contains the least number of layers. On the other hand, the Gap Regularizer allowed SFCN to obtain the overall best results on the RHC dataset and better GAME on the MOT17-09 dataset. However, the impacts of the Gap Regularizer are always statistically insignificant on ResNet-101.

**Table 4:** Number of parameters and depth of all backbone networks provided by the C-3-Framework.

Backbone network	Number of parameters	Depth
Resnet-50	8,674,625	102 layers
Resnet-101	27,666,753	221 layers
CSRNet	16,263,489	36 layers
SFCN	38,596,801	237 layers

Although the SFCN model’s base architecture is that of ResNet-101, one difference between them is the existence of “back-end” features which may impact the regularizer’s significance. These back-end features refer to the layers in the CNN models where the width of the layers are inversely proportional to the depth of the model. These layers are placed after the final convolution block of the model. Following the results explained above where the differences in the evaluation results of ResNet-101 are generally statistically insignificant unlike that of SFCN, it can be inferred that the Gap Regularizer can also yield significant impact by reducing the errors on deeper models with back-end features such as SFCN.

## Conclusion

In this article, we have described an approach named Gap Regularizer, which is aimed to allow a deep-learning-based people counting model to count only people inside an RoI. Through the experiment, we proved that Gap Regularizer can improve the performance of ResNet-50, CSRNet, and SFCN to regress counting density maps with annotations only for the people inside the RoI, hinting that smaller models and deeper models with back-end features can benefit from it. Most of their improvements were statistically tested as significant with *P*-values less than 0.05 from the results of the Mann-Whitney U test. One notable finding is that the regularizer is capable of drastically improving the models’ output SSIM, proving that it allows the models to better distinguish the crowd inside and outside of the RoI. To further improve our work in the future, a promising direction is to design an architecture that can integrate the supervision of inside and outside people in a more collaborative learning strategy. This approach can potentially deliver better performance than the regularizer that we proposed in this study.

## Supplemental Information

10.7717/peerj-cs.1067/supp-1 Supplemental Information 1 Code.

## Additional Information and Declarations

### Data Availability

The following information was supplied regarding data availability: Data is available at Mendeley: Pardamean, Bens; Muljo, Hery Harjono; Abid, Faizal; Herman, Herman; Susanto, Albert; Cenggoro, Tjeng Wawan (2020), “RHC (Room Human Counting)”, Mendeley Data, V1, DOI 10.17632/vt5c8h6kmh.1.
